# ToxR of *Vibrio cholerae *affects biofilm, rugosity and survival with *Acanthamoeba castellanii*

**DOI:** 10.1186/1756-0500-5-33

**Published:** 2012-01-16

**Authors:** Soni P Valeru, Sun N Wai, Amir Saeed, Gunnar Sandström, Hadi Abd

**Affiliations:** 1 Karolinska Institute, Department of Laboratory Medicine, Division of Clinical Microbiology, Karolinska University Hospital, Huddinge, SE-141 86, Stockholm, Sweden; 2 Department of Molecular Biology, Umeå University, 90187 Umeå, Sweden

**Keywords:** *V. cholerae*, Outer membrane proteins, Rugose colonies, Biofilm, Association with amoebae

## Abstract

**Background:**

*Vibrio cholerae *causes the diarrheal disease cholera and utilizes different survival strategies in aquatic environments. *V. cholerae *can survive as free-living or in association with zooplankton and can build biofilm and rugose colonies. The bacterium expresses cholera toxin (CT) and toxin-coregulated pilus (TCP) as the main virulence factors. These factors are co-regulated by a transcriptional regulator ToxR, which modulates expression of outer membrane proteins (OmpU) and (OmpT). The aims of this study were to disclose the role of ToxR in expression of OmpU and OmpT, biofilm and rugose colony formation as well as in association with the free-living amoeba *Acanthamoeba castellanii *at different temperatures.

**Results:**

The *toxR *mutant *V. cholerae *produced OmpT, significant biofilm and rugose colonies compared to the wild type that produced OmpU, decreased biofilm and did not form rugoes colonies at 30°C. Interestingly, neither the wild type nor *toxR *mutant strain could form rugose colonies in association with the amoebae. However, during the association with the amoebae it was observed that *A. castellanii *enhanced survival of *V. cholerae *wild type compared to *toxR *mutant strain at 37°C.

**Conclusions:**

ToxR does seem to play some regulatory role in the OmpT/OmpU expression shift, the changes in biofilm, rugosity and survival with *A. castellanii*, suggesting a new role for this regulatory protein in the environments.

## Background

*Vibrio cholerae *is a gram-negative bacterium causing severe diarrhoeal disease cholera in Asia, Africa, and America. Many millions of cholera cases occurred endemically and pandemically worldwide [1].

*V. cholerae *adopts several survival strategies in aquatic environments. The bacterium can survive as free-living or in association with phytoplankton/zooplankton, crustaceans, and molluscs in coastal and estuarine environments [2]. *V. cholerae *forms biofilms on biotic and abiotic surfaces, thereby protecting themselves with this exopolymer barrier [3], biofilm can provide protection from toxic compounds, such as antibiotics, thermal stress, and predation [4]. The bacterium has been described to switch between the smooth and rugose colony morphotypes contributing to its environmental survival. The exopolysaccharide (EPS) materials of rugose colony-forming *V. cholerae *strains were recognized as a heavy, fibrous, electron-dense, ferretin-stained layer surrounding the cells, but smooth colony *V. cholerae *did not appear to have this EPS layer surrounding it [5]. The cell surface EPS materials confer a rugose colony morphology and resistance to osmotic and oxidative stresses. The regulation of EPS synthesis in bacteria is complex and involves multiple systems utilizing both positive and negative regulation [5].

Recent studies have shown that *V. cholerae *has developed a new survival strategy to grow and survive inside free-living amoeba *Acanthamoeba castellanii *[6-10].

*V. cholerae *expresses cholera toxin and toxin-coregulated pilus as the main virulence factors, which are co-regulated by a transcriptional regulator ToxR. ToxR, however, independently of the transcriptional activators TcpP and ToxT, modulates expression of two outer membrane proteins OmpU and OmpT. Transcription of *ompU *is induced by ToxR, whereas transcription of *ompT *is repressed by ToxR [11,12].

In this paper we study the effect of ToxR regulatory protein in expression of OmpU and OmpT, biofilm and rugosity as well as survival of *V. cholerae *O1 with *A. castellanii *by constructing a *toxR *deletion mutant.

## Methods

### Bacterial strains and plasmids used in this study

The bacterial strains and plasmids used in this study are listed in Table 1. Strains were grown in LB medium at 37°C. Strains were stored frozen in LB medium with 15% glycerol at -80°C. Antibiotics were used at the following concentrations: carbencillin 50 μg/mL, streptomycin 50 μg/ml, kanamycin 50 μg/mL.

**Table 1:** Bacterial strains and plasmids used in this study

Strain or plasmid	Description	Reference
***V. cholerae ***O1 E1Tor Inaba	Strain A1552, Wild type	[13]

***V. cholerae ***Δ***toxR***	Transcriptional activator deletion mutant of Strain A1552	This study

*E. coli *DH5α	Φ80d*lac*ZΔM15 *recA1gyrA96 thi-1 hsdR*17 (r_k_^- ^m_k_^+^) *supE44*	[14]

Sm10λpir	*relA1deoR*Δ*(lacZYA-argF)U169*	[15]

	*thi-1, thr, leu, tonA, lacY, supE*,	

	*recA*::Rp4-2-Tc::Mu,Km^R^,(λpir)	

Plasmid Puc 18	Ap^R ^lacZaovi colE1	[16]

Plasmid PCVD442	*oriR6K mobRP4 sacB *Ap^r^	this study

### Growth conditions

Luria-Bertani (LB) media, All strains were grown in LB medium (1% Bacto Tryptone, 0.5% yeast extract, 0.5% NaCl) with appropriate selection. For antibiotic selection, 50 μg/mL carbenicillin or 50 μg/mL streptomycin was used.

### Construction of the internal in-frame toxR deletion mutant

To introduce a deletion at the chromosomal toxR locus we used a double cross over procedure. A plasmid pCVD442 was used as a suicidal vector. In the first step two different asymmetric polymerase chain reactions (PCR) amplified with primers toxR-A (5'CCC ATC CAC TAA ACG GTA CCA TCC GAA CAT CTA ATG TCC CAG-3') toxR-B (5'-GCG TCT AGA ACT CTT TAC CTT CTT CAC GCAG-3') toxR-C (5'-GCG TCT AGA ATC TTC GCT GAA GGT ATG CAGT-3') and toxR-D(5'TGG TAC CGT TTA GTG GAT GGG GCC ATC AAA GTG TGT GAG TAG-3') were used to generate fragments upstream and downstream of the sequences targeted for deletion. In the second step, the upstream and downstream fragments were annealed at their overlapping region and amplified by PCR as a single fragment, using the outer primers (toxR-A and toxR-D). The PCR products were phenol-chloroform extracted, ethanol precipitated, washed with 70% ethanol, vacuum dried, resuspended in 50 mL of Xba-I restriction buffer containing 40 U of *XbaI *restriction enzyme, and digested overnight at 37°C. The DNA fragments were gel purified, ligated into *XbaI *digested and alkaline phosphatase-treated pCVD442 and then, electroporated into *E. coli *SM10λpir. The mutant constructs were checked by PCR and sequencing. The confirmed deletion constructs were introduced into *V. cholerae *by conjugation, screened on 15% sucrose containing plates, and the carbenicillin sensitive clones were assessed for double cross-over recombination. The recombinant colonies were checked by PCR.

### Expression of outer membrane proteins

Bacterial cultures were grown overnight in 2 ml LB media at 37°C. The entire contents were centrifuged at 18,000 × g for 15 min. The supernatants were collected in new tubes and the pellet was suspended in 100 μl sample buffer and boiled for 10 min. The supernatant samples were precipitated with 10% TCA for 30 min on ice. The samples were centrifuged at 18,000 × g for 30 min and washed with 80% acetone. The pellet was resuspended in standard SDS-PAGE sample buffer. Samples were analysed by 13% SDS-PAGE.

### Biofilm analysis

*V. cholerae *wild type and *toxR *mutant strains were grown on LB agar plates overnight at 37°C. Few colonies from each strain were suspended in 20 mL LB broth and incubated in shaking incubator until the absorbance reached 0.6 units at 600 nm. 5 mL of 1:100 dilution of this suspension was transferred into triplicated glass tubes (13 by 100 mm) and incubated for 18 h at 30°C. Representative biofilm formed by wild type and *toxR *mutant strains was visualised photographically and the tubes were rinsed with distilled water then filled with 1% crystal violet stain. After 15 min, the tubes were rinsed and the biofilm-associated crystal violet in the tubes was suspended with 95% ethanol. The absorbance of the resulting suspension was measured by spectrophotometry at 570 nm to quantify the biofilm formation.

### Colony morphology

For colony morphology assay, *V. cholerae *wild type and *toxR *mutant strains were grown on LB agar plates for 1 day at 30°C and left at room temperature until appearance of rugose colonies after 6 days. Percentage of rugose colonies was estimated and representative smooth as well as rugose colonies were visualized photographically.

### Co-cultivation assay

The co-cultivation assay was based on a method presented previously. Axenically maintained amoebae were grown at 30°C to a final concentration of 2 × 10^5 ^CFU/mL in ATCC medium as described above. Co-cultivations of *V. cholerae *with *A. castellanii *were incubated in NUNC tissue culture flasks (75 cm) purchased from VWR International (Stockholm, Sweden) filled with 50 mL ATCC medium 712 containing *A. castellanii *at a concentration of 2 × 10^5 ^CFU/mL and the particular *V. cholerae *species at a concentration of 2 × 10^6 ^CFU/mL. Control flasks containing bacteria or amoebae only were prepared in the same way and with the same initial concentration as the co-culture flasks. All flasks were triplicates and incubated at 25°C and 37°C. Samples were taken and plated on blood agar plates regularly to study growth and survival of *V. cholerae*.

## Results

### Construction of a Δ *toxR *in *V. cholerae *and expression of outer membrane proteins

**Figure 1:**
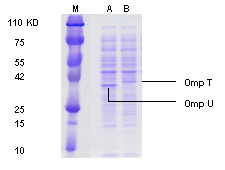
**Outer membrane proteins expression by SDS-PAGE gel from whole cell lysates**. Lanes M, A and B represent marker, *V. cholerae *wild and toxR mutant strain. The gel confirmed that the wild *V. cholerae *expressed OmpU as it is usually activated by ToxR (A) while the constructed toxR mutant expressed OmpT (B).

An internal in-frame *toxR *deletion mutant in *V. cholerae *was constructed as described in the materials and methods and examined for expression of outer membrane proteins by SDS-PAGE. The result showed that the *V. cholerae toxR *mutant strain expressed OmpT compared to the wild type strain, which expressed OmpU (Figure 1)

### Biofilm analysis

To study the effect of ToxR in biofilm formation at 30°C in LB broth, the biofilm and biofilm-associated crystal violet formed by the wild type *V. cholerae *or the *toxR *mutant strains was visualised photographically and absorbance of the biofilm-associated crystal violet ethanol solution was measured spectrophotometrically.

**Figure 2:**
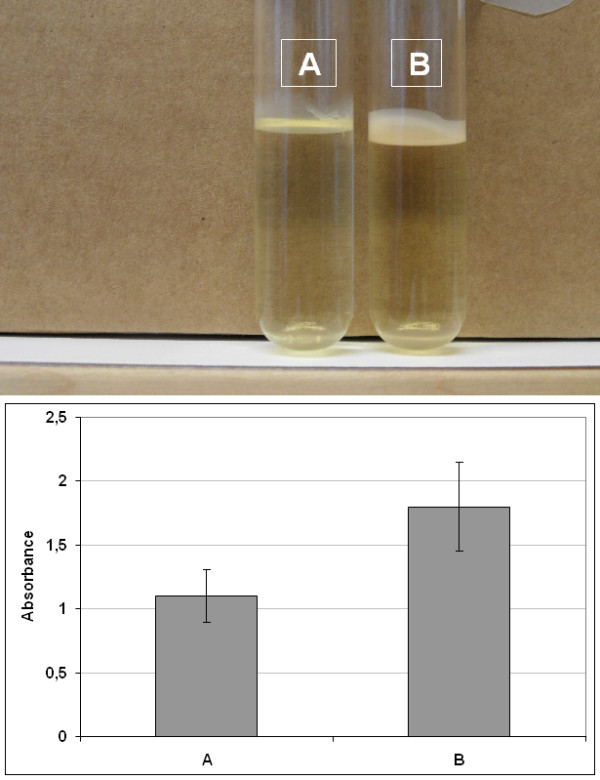
**Biofilm formation**. Upper panel showed representative photographs of biofilms formed by wild type *V. cholerae *(A) and *toxR *mutant strain (B). Lower panel showed representative measurement of the absorbance, which is directly proportional to the concentration of biofilm-associated crystal violet formed by wild type *V. cholerae *(A) and *toxR *mutant (B) strains. Data indicates mean ± SD of three independent experiments.

The photographical analysis showed that the wild type *V. cholerae *(Figure 2A upper panel) had repressed biofilm formation compared to the *toxR *mutant strain (Figure 2B upper panel). Moreover, it was found that the absorbance of the biofilm produced by the wild type *V. cholerae *(Figure 2A lower panel) and the *toxR *mutant strain (Figure 2B lower panel) were 1.1 ± 0.2 and 1.8 ± 0.35, respectively. However, the spectrophotometry showed that the *toxR *mutant of *V. cholerae *had enhanced biofilm formation, which was confirmed by the statistical analysis, since the difference in absorbance was statistically significant (*p *of *t*-test was < 0.05).

### Colony morphology

**Figure 3:**
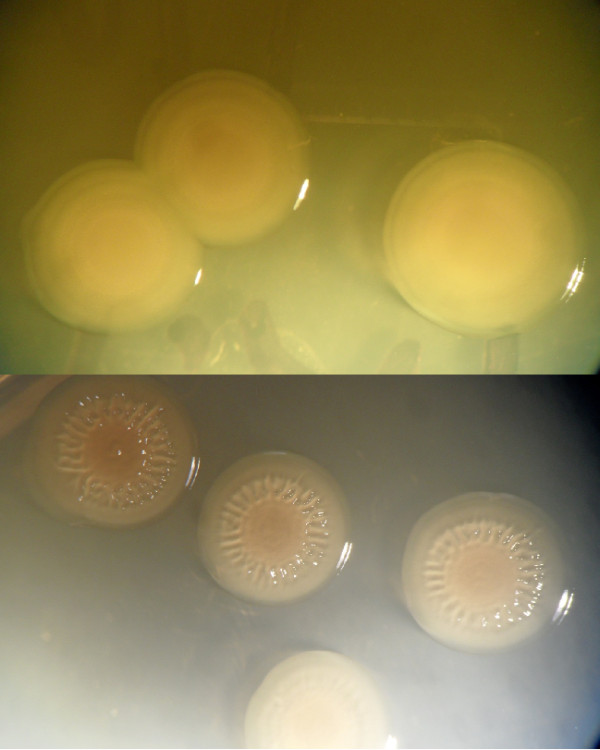
**Colony morphology of *V. cholerae *strains**. Upper panel represents smooth colonies of wild type strain. Lower panel represents rugose colonies of *toxR *mutant strain.

The result showed that all colonies of *V. cholerae *wild type strain were smooth since this strain did not form any rugose colony (Figure 3 upper panel). However, it was found that 35 ± 10% of *toxR *mutant strain colonies was rugose (Figure 3 lower panel) and 65 ± 10% was smooth. The ability of each strain to form rugose colonies was significantly differed by χ2 test (*p *< 0.001).

### Effect of *ToxR *protein on growth, survival and rugose colony formation of *V. cholerae *associated with *Acanthamoeba castellanii*

Rugose colony formation, growth and survival of 2.0 ± × 10^6 ^cell/mL *V. cholerae *wild type and *toxR *mutant cultivated in ATCC medium were compared with those of the same strains cultivated with 2.0 × 10^5 ^cell/mL *A. castellanii *in the same medium.

**Figure 4:**
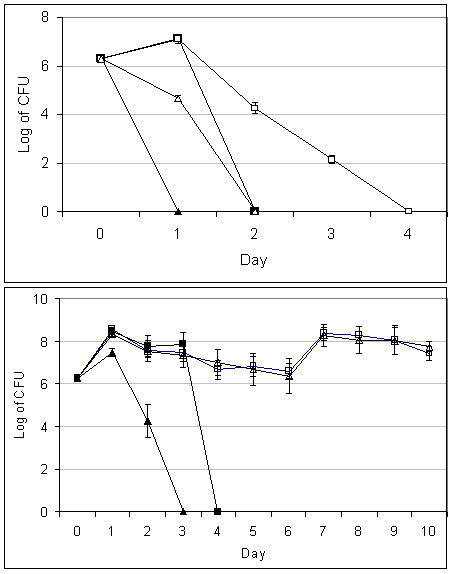
**Growth and survival of *V. cholerae *strains in absence and presence of *A. castellanii *at 37°C upper panel and at 25°C lower panel**. Alone cultivated wild type *V. choleare *(■) and cultivated with *A. castellanii *(□). Alone cultivated *toxR *mutant strain (▲) and cultivated with *A. castellanii *(Δ). Data indicates mean ± SD of three independent experiments.

The result showed that the *V. cholerae *wild type and the *toxR *mutant survived differently at different temperatures. At 37°C the wild type strain grew to 1.3 × 10^7 ^± 5.8 × 10^6 ^CFU/mL and survived 1 day in the absence of amoebae compared to 1.4 × 10^2 ^± 5.5 × 10 CFU/mL and survived 3 days in the presence of the amoebae, whereas the *toxR *mutant strain died on the first day in the absence of amoebae but survived 1 day to 4.7 × 10^5 ^± 4.0 × 10^5 ^CFU/mL in the presence of amoebae (Figure 4 upper panel).

At 25°C the wild type strain grew to 7.5 × 10^7 ^± 1.9 × 10^7 ^CFU/mL and survived 3 days, in the absence of amoebae compared to the *toxR *mutant strain, which decreased to 2.0 × 10^4 ^± 1.0 × 10^4 ^CFU/mL and survived 2 days. Whereas in the presence of the amoebae, the wild type and the mutant strains grew to 3.3 × 10^7 ^± 2.6 × 10^7 ^CFU/mL and 6.0 × 10^7 ^± 3.0 × 10^7 ^CFU/mL and survived 10 days, respectively (Figure 4 lower panel).

During the cultivation with the amoebae it was observed that the wild type and the *toxR *mutant strains did not form any rugose colonies at both 25°C and 37°C.

## Discussion

*V. cholerae *adopts several survival strategies in aquatic environments. The bacterium can survive as free-living or in association with zooplankton and can build biofilm and rugose colonies [2,3]. Recent studies have shown that *V. cholerae *has an enhanced growth in association with the free-living amoeba *A. castellanii *at 30°C [6-10] and both these microorganisms are detected in same water samples from cholera endemic area [17].

In this study, the internal in-frame *toxR *deletion mutant *V. cholerae *strain succeeds to express the ToxR-repressed OmpT instead of the ToxR-activated OmpU. This altered expression of the ToxR-regulated OmpU and OmpT was confirmed by previous study and might affect the viruelence of *V. cholerae *[11].

Our paper has disclosed the role of ToxR regulatory protein in the environmental survival strategies of *V. cholerae *such as biofilm formation, switching from smooth to rugose colony morphotypes and association with the free-living amoeba namely *A. castellanii*.

Our results demonstrate that ToxR clearly affects biofilm and rugose formation since the differences in biofilm and rugose colony formation between *V. cholerae *wild type and *toxR *mutant were significantly high by *t*-test and χ2 test, respectively.

Effect of ToxR on growth and survival of *V. cholerae *associated with *A. castellanii *was studied by viable count performed on blood agar. *V. cholerae *wild type strain survived longer than the *toxR *mutant strain at 37°C, in the presence or absence of the amoebae. Interestingly, the association with *A. castellanii *enhanced survival of both bacterial strain but the wild type strain survived longer uncovering a role of ToxR in the survival of *V. cholerae *associated with protozoa in aquatic environment.

In cultivation with the amoebae at 25°C, it was observed that both *V. cholerae *wild type and *toxR *mutant strains survived more than 10 days.

Despite *V. cholerae *wild type and *toxR *mutant strains survived more than 10 days in cultivation with the amoebae at 25°C, these strains did not form any rugose colony indicating that the bacteria avoided starvation. However, presence of the amoebae might enrich the cultivation medium and viable count of the bacteria was performed on enriched plates (blood agar plates).

Analogies to rugosity can be found in a number of other bacterial species, including the expression of alginate by mucoid strains of *Pseudomonas aeruginosa *and the expression of an adhesive EPS by members of the marine genus [5].

Spontaneous and reversible variation in cell-associated and cell-free EPS production represents an optimal adaptive mechanism that facilitates survival in stressful environments [18].

Although, over 20 genes are co-ordinately controlled by the ToxR regulon [19], the mechanism of switching is currently under study and it seemed to be regulated by exopolysaccharide related phase variation. Phase variation can occur via DNA inversion, DNA recombination, and slipped strand mispairing and is known to be involved in controlling the expression of several surface structures of gram-negative bacteria, including fimbriae, flagella, outer membrane proteins, lipopolysaccharide, and capsular polysaccharide [20]. However, role of ToxR was found to be critical for *V. cholerae *bile resistance, virulence factor expression, and intestinal colonization [11,12]. Surprisingly, ToxR homolog from *V. anguillarum *was found to regulate its own production, bile resistance, and biofilm formation [21] which might emphasis the regulatory role of ToxR in the expression of virulence factors for *Vibrio *species.

## Conclusions

Our results demonstrate that ToxR does seem to play some regulatory role in the OmpT/OmpU expression shift and in the environmental survival strategies of *V. cholerae *such as biofilm formation, switching from smooth to rugose colony and association with the free-living amoeba *A. castellanii*, suggesting a new role for this regulatory protein.

## Authors' contributions

SV carried out the construction of deletion, outer membrane detection, the analysis of biofilm-and co-cultivation assay and drafted the manuscript. SW contributed to providing of bacterial strains, guiding and design of deletion, and critical reading. AS participated in writing. GS participated in writing and critical reading. HA conceived the study, designed co-infection assay, performed the statistical analysis and helped to draft the manuscript. All authors read and approved the final manuscript.

## Competing interests

The authors declare that they have no competing interests.
